# Dietary and smoking habits during the exam period and their effect on the academic achievement among Syrian medical students

**DOI:** 10.1186/s12909-023-04950-6

**Published:** 2024-01-12

**Authors:** Anas Bitar, Fady Barakat, Angie Hawat, Bayan Alsaid

**Affiliations:** 1 Faculty of Medicine, Damascus University, Damascus, Syria; 2 Laboratory of Anatomy, Faculty of Medicine, Damascus University, Damascus, Syria

**Keywords:** Academic achievement, Dietary habits, Eating habits, Drinking habits, Smoking habits, Medical students, Syria

## Abstract

**Background:**

Dietary habits are crucial for maintaining overall health and have been shown to impact academic performance. However, little research has been conducted on the specific eating, drinking, and smoking habits during the exam period and their effect on academic performance. Therefore, this study aimed to investigate dietary and smoking habits and their relationship with academic performance among medical students at Damascus University. Findings from this study can inform interventions to improve both dietary habits and academic performance.

**Methods:**

A cross-sectional design was used to investigate the association between dietary habits during the exam period and academic performance among 608 medical students. Data were collected using a self-administered paper-based questionnaire that was randomly distributed across multiple classes. The statistical analysis employed Spearman’s rank correlation and multiple linear regression to examine the relationship between the different amounts of diet behaviour during the exams and the reported average students’ grades from the last two semesters. The regression model was adjusted for age, sex, residency type, and residency partners.

**Results:**

In both Spearman's rank correlation and multiple linear regression analyses, we observed consistent negative associations between specific dietary habits and self-reported average grades. These included daily consumption of tea (B = -0.334, *p* = 0.022), instant coffee (B = -0.682, *p* = 0.001), and weekly fast food consumption (B = -0.583, *p* = 0.038). Notably, traditional coffee was found to be significant in the Spearman analysis but did not exhibit significance in the regression analysis. Furthermore, the regression analysis revealed a negative association between water pipe consumption and academic performance (B = -0.700, *p* = 0.049). No significant association was observed between other dietary habits, cigarette smoking, and academic performance.

**Conclusions:**

The study highlights a negative, but weak, association between academic achievement and specific dietary habits during the exam period. However, further longitudinal studies are needed to establish causal relationships. Additionally, the study suggests exploring the impact of other habits such as study and sleeping habits, which may have a more significant impact on academic achievement.

**Supplementary Information:**

The online version contains supplementary material available at 10.1186/s12909-023-04950-6.

## Background

Eating habits refer to the repeated and purposeful behaviours of individuals in selecting, consuming, and utilizing specific foods or diets, which are influenced by cultural and social factors [1]. Dietary habits have long been recognized as a crucial factor in maintaining overall health and well-being [2–4]. Furthermore, the dietary habits of university students and their relation with academic performance are considered to be the main topic of many studies [5–8]. These studies have indicated that diets rich in processed and unhealthy foods, which are low in essential nutrients, are associated with lower academic performance. Conversely, adopting a healthy diet has been found to be positively correlated with better academic outcomes. In addition to dietary habits, certain studies have examined the impact of smoking and alcohol consumption on academic performance [9, 10].

Despite the growing body of research on this topic [11], little is known about the specific eating, drinking, and smoking habits during the exam period and their impact on the academic performance of medical students, particularly in medical schools where the exam period may span over a prolonged period of time (1–2 months).

This study was carried out to obtain information on the prevalence of eating, drinking, and smoking habits during the exam period and their relation to the academic performance of medical students at Damascus University. It is expected that understanding the potential association between dietary intake and academic performance will provide valuable information that can be used to develop interventions and programs aimed at improving students’ dietary habits and academic outcomes.

## Methods

### Sampling, data collection, and ethical considerations

Between May 26, 2022, and June 5, 2022, a cross-sectional study was conducted using a paper-based, self-administered anonymous questionnaire to collect data. The study was reviewed and approved by the Institutional Review Board (IRB) in the Faculty of Medicine at Damascus University, and all participants provided written consent. The study's sample size was calculated using Taro Yamane's formula [12], which is represented as follows:

$$\mathrm{n }=\mathrm{ N}/\left(1 +{\text{N}}{\left({\text{e}}\right)}^{2}\right)$$

Here, n represents the desired sample size, N is the total population (5,537 in this case), and e stands for the level of precision, margin, or sampling error (in this case, assumed as 0.05, which is equivalent to a ± 5% margin) at a 95% confidence level. By applying this formula to the specific context of the study we get a sample size of 374. However, we aimed for a higher number (900).

The inclusion criteria encompassed undergraduate students from Damascus University, Faculty of Medicine, excluding first-year students (preparatory year). Participants were required to have maintained continuous enrolment in the university over the last two semesters. We implemented a systematic approach to select our aimed sample of 900 students, ensuring an equal representation across different academic years. To attain this goal, we initiated the sampling process by dividing the total required sample size by the number of academic years. This initial step helped us determine the target number of students to include from each year.

Subsequently, we proceeded to encompass all available practical classes within each academic year as part of our sampling framework. To calculate the number of classes to be included from each year, we divided the target number of students for that year by the average class attendance, which was typically around 30 students per class, and then the selection of classes was randomized through the use of an Excel function. We enrolled all the students from each selected class to ensure comprehensive data collection. In addition to that, we allocated 5% of the sample size to students who did not attend practical classes but were located elsewhere within the college campus. It is important to note that these students were not chosen at random. Instead, we approached and included those individuals who were available and willing to participate. In line with our commitment to data quality, we kindly requested that students refrain from completing the questionnaire if they had previously done so. Furthermore, it's crucial to emphasize that students are required to attend their respective practical classes, and each group of students follows a set class schedule. This strict adherence to a standardized class schedule greatly facilitated our data collection process. Students voluntarily participated in the study and had the option to decline participation. Only students with completed questionnaires were included in the study.

### Survey design

We designed the questionnaire in Arabic after conducting a comprehensive literature review and considering Syrian cultural habits. This included considering popular practices such as the consumption of traditional beverages like mate, as well as the cultural prevalence of water pipe, which is known for its popularity in the region. The survey was mainly adopted from previously published studies [5, 13, 14]. It consisted of three parts: (1) Student characteristics, (2) eating and drinking habits during the exam period, and (3) smoking habits during and outside the exam period. An English version of the questionnaire can be found in the supplementary materials. Students were asked to report their average grades from the last two semesters as a measure of academic performance.

### Pilot testing

A pilot test of the questionnaire was conducted among a random group of 42 students. We assessed face validity through participant feedback, making necessary adjustments to enhance the questionnaire’s clarity, length and relevance. However, these students were not included in the main study.

### Data analysis

Data were analyzed using SPSS v.26.0 (SPSS Inc., Chicago, IL, USA). Quantitative variables were reported as means (± SD), and qualitative variables were reported as frequencies and percentages. Spearman’s rank correlation was used to investigate the relationship between self-reported average grades from the last two semesters and rates of consumption of foods, drinks, water pipe, and cigarettes. Independent t-tests were used to analyze the variables of sex and scheduled eating.

To further assess the independent effect of each variable on students' marks while controlling for potential confounding factors, a multiple linear regression analysis (Enter Method) was conducted. The dependent variable was the students’ average grades from the last two semesters. The independent variables of interest were all the reported dietary and smoking habits during the exam period. Covariates included age, sex, type of residency, and residency partners. Multicollinearity was assessed using the Variance Inflation Factor (VIF). A significance level of 0.05 was used to determine statistical significance.

## Results

### Study population

Out of the 672 students who initially responded, a total of 608 medical students successfully completed the questionnaire. The mean age of the participants was 21.5 ± 1.47. The average reported grade was 83.33 ± 5.51. Most of the students lived in owned houses (397, 65.3%), while 120 (19.7%) lived in rented houses, and only 91 (15%) lived in dormitories. The majority of the students lived with their family (429, 70.6%), while only 34 (5.6%) lived alone, and 13 (2.1%) lived with their relatives. Other study population characteristics are shown in Table 1. To compare the mean scores between males and females, an independent samples t-test was performed, which revealed no significant difference (83.1 ± 5.54 for females vs. 83.6 ± 5.49 for males, t = -1.118, *p* = 0.26).

**Table 1:** Characteristics of the study population (*n* = 608)

Students Characteristics	n (%) or mean ± SD
Sex	
Male	286 (47)
Female	322 (53)
Age	21.5 ± 1.47
Grades (%)	83.33 ± 5.51
Year of study	
2nd	120 (19.7)
3rd	137 (22.5)
4th	153 (25.2)
5th	138 (22.7)
6th	60 (9.9)
Residency type	
Dormitory	91 (15)
Rented house	120 (19.7)
Owned house	397 (65.3)
Residency partners	
Alone	34 (5.6)
Friends/Roommates	95 (15.6)
Relatives	13 (2.1)
Brothers/Sisters	37 (6.1)
Family	429 (70.6)

### Eating and drinking habits during exams

Regarding daily consumption, 218 (35.9%) students did not consume traditional coffee, and 37 (6.1%) drank 5 or more cups daily. In addition, 243 (40%) students did not consume instant coffee. As for tea and mate, 180 (29.6%) and 386 (63.5%) students did not consume any cups daily, respectively.

In terms of weekly consumption, most of the students did not consume energy drinks (583, 95.9%). Furthermore, 395 students (65%) had breakfast daily. Nearly half of the students (333, 54.8%) did not drink any cup of natural fruit juice weekly, and around half of them (324, 53.3%) rarely consumed fast food weekly. Additionally, 329 students (53.6%) reported having scheduled eating habits (breakfast, lunch, dinner). Details about diet consumption during the exams and the corresponding students’ average grades from the last two semesters are provided in Table 2.

**Table 2:** Diet consumption during the exams and corresponding students’ average grades from the last two semesters (*n* = 608)

Item	Daily Consumption n (%)	Students’ grades	Item	Daily Consumption n (%)	Students’ grades
Traditional coffee (cups)			Tea (cups)		
0	218 (35.9)	84.06 ± 5.53	0	180 (29.6)	83.87 ± 4.99
1	110 (18.1)	83.23 ± 6.00	1	138 (22.7)	84.33 ± 5.44
2	121 (19.9)	83.64 ± 5.46	2	136 (22.4)	82.57 ± 5.77
3	78 (12.8)	81.79 ± 5.02	3	67 (11)	83.06 ± 5.98
4	44 (7.2)	82.70 ± 4.51	4	29 (4.8)	82.17 ± 5.14
5 or more	37 (6.1)	82.34 ± 5.60	5 or more	58 (9.5)	81.96 ± 5.81
Instant coffee (cups)			Mate (cups)		
0	243 (40)	84.26 ± 5.19	0	386 (63.5)	83.29 ± 5.47
1	207 (34)	83.45 ± 5.27	1	54 (8.9)	82.6 ± 5.63
2	99 (16.3)	82.17 ± 5.59	2	54 (8.9)	83.66 ± 6.41
3	32 (5.3)	80.83 ± 6.88	3	34 (5.6)	83.72 ± 6.29
4	15 (2.5)	81.25 ± 4.96	4	19 (3.1)	83.96 ± 5.71
5 or more	12 (2)	81.35 ± 7.90	5 or more	61 (10)	83.53 ± 4.35
**Item**	**Weekly Consumption n (%)**	**Students’ grades**	**Item**	**Weekly Consumption n (%)**	**Students’ grades**
Energy drinks (beverages, 250 ml)			Natural fruit juice (cups)		
0	583 (95.9)	83.37 ± 5.43	0	333 (54.8)	83.41 ± 5.11
1–3	24 (3.9)	82.36 ± 7.37	1–3	250 (41.1)	83.41 ± 5.75
4 or more	1 (0.2)	83.00	4–6	18 (3)	80.98 ± 8.2
			7 or more	7 (1.2)	83.07 ± 7.24
Fruit			Fast food (meals)		
Rarely	158 (26)	82.91 ± 5	Rarely	324 (53.3)	83.91 ± 5.37
1–2	250 (41.1)	83.66 ± 5.51	1–2	189 (31.1)	82.99 ± 5.33
3–4	113 (18.6)	83.2 ± 6.50	3–4	61 (10)	82.71 ± 6.51
Daily	87 (14.3)	83.34 ± 5.05	Daily	34 (5.6)	80.88 ± 5.3
Breakfast (meals)			Snacks (meals)		
0	40 (6.6)	82.57 ± 5.08	Rarely	111 (18.3)	84.28 ± 4.87
1	33 (5.4)	82.48 ± 6.71	1–2	215 (35.4)	82.72 ± 5.42
2	34 (5.6)	83.89 ± 5.32	3–4	127 (20.9)	83.56 ± 6.01
3	31 (5.1)	82.43 ± 5.21	Daily	155 (25.5)	83.31 ± 5.61
4	26 (4.3)	81.86 ± 4.89	Scheduled eating (breakfast, lunch, dinner)		
5	21 (3.5)	83.22 ± 5.33	Yes	326 (53.6)	83.41 ± 5.77
6	28 (4.6)	85.60 ± 5.09	No	282 (46.4)	83.25 ± 5.21
7	395 (65)	83.45 ± 5.54			
Alcohol (beverages)			Vit. C (tablets)		
0	566 (93.1)	83.23 ± 5.48	0	440 (72.4)	83.34 ± 5.45
1	27 (4.4)	84.37 ± 5.00	1–3	130 (21.4)	83.33 ± 5.51
2–3	9 (1.5)	84.15 ± 8.03	4–6	26 (4.3)	82.83 ± 6.63
4–6	4 (0.7)	89.75 ± 5.4	7–10	6 (1.0)	83.86 ± 4.32
7 or more	2 (0.3)	82.50 ± 6.36	11 or more	6 (1.0)	84.00 ± 7.56

### Smoking habits

**Fig. 1:**
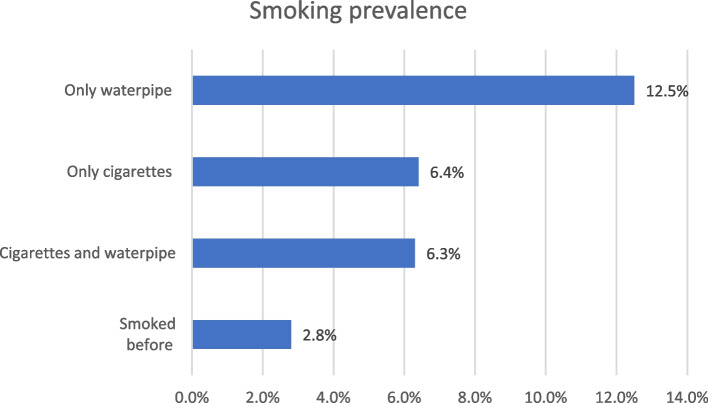
Prevalence of smoking among participants (*n* = 608)

**Fig. 2:**
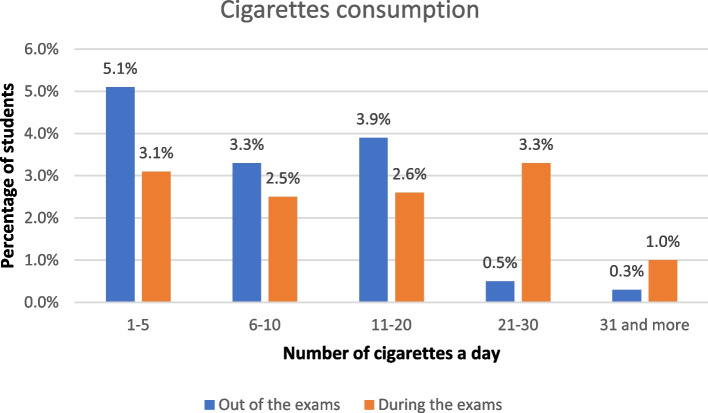
Comparison of cigarette consumption percentage during and outside the exam period (*n* = 608)

**Fig. 3:**
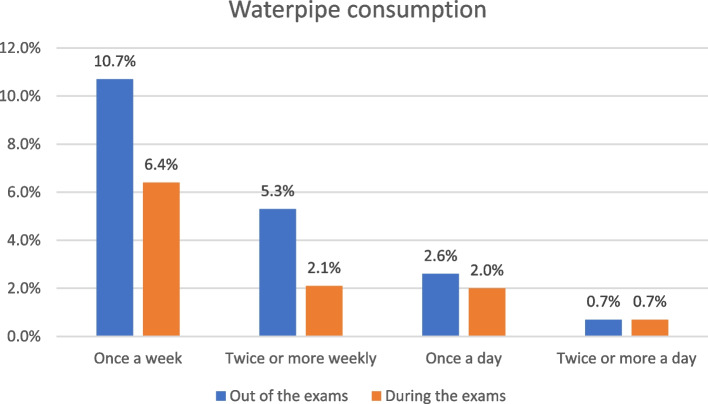
Comparison of waterpipe consumption percentage during and outside the exam period (*n* = 608)

The prevalence of smoking among participants was investigated with respect to cigarettes and water pipe. Out of the total sample (608), 39 students (6.4%) reported only smoking cigarettes, 76 (12.5%) smoked only water pipe, and 38 (6.3%) smoked both. Moreover, 17 (2.8%) participants reported having smoked in the past (Fig. 1). The number of smokers was found to be lower during the exam period than outside the exam period for both cigarettes (80 outside the exam vs. 76 during the exam) and water pipe (117 outside the exam vs. 68 during the exam). However, the percentage of students who smoked 21 or more cigarettes per day was higher during the exam period (4.3%) than at other times (0.8%). Figures 2 and 3 display the comparison of the amount of smoking during and outside the exam period.

### Exam period habits and reported grades

In order to examine the relationship between students' eating and drinking habits during the exam period and their grades, Spearman's rank correlation and multiple linear regression were employed. Our findings revealed a significant, but weak, negative correlation between students' grades and their daily consumption of traditional coffee (r_s_ = -0.122, *p* = 0.003), tea (r_s_ = -0.115, *p* = 0.005), and instant coffee (r_s_ = -0.168, *p* < 0.001). However, this negative correlation was not observed for mate (r_s_ = 0.019, *p* = 0.64), energy drinks, natural fruit juice, and other drinks. Additionally, fast food consumption was found to be significantly and negatively correlated with students' grades (r_s_ = -0.117, *p* = 0.004), while no significant correlation was found for snacks (r_s_ = -0.031, *p* = 0.45), or weekly breakfast consumption (r_s_ = 0.033, *p* = 0.41), or weekly fruit consumption (r_s_ = -0.026, *p* = 0.52). Furthermore, independent samples t-test was utilized to examine the potential effect of having scheduled eating habits (breakfast, lunch, and dinner) on academic performance. However, no significant effect was observed (t = -0.356, *p* = 0.72). In relation to smoking habits during the exam period, there was no significant correlation between the amount of smoking and students' grades for both cigarettes and water pipe (r_s_ = -0.026, *p* = 0.53) and (r_s_ = -0.067, *p* = 0.10), respectively. Table 3 demonstrates the relationship between different types of diet habits with self-reported students’ average grades using Spearman’s rank correlation.

**Table 3:** Relationship between different types of dietary habits and self-reported students’ average grades using Spearman’s rank correlation (*n* = 608)

Eating/smoking habit during exams	Variable description and frequency	Test results	*P*
Traditional coffee	Number of times items consumed per day (categorical data)	r_s_ = -0.122	0.003*
Tea	Number of times items consumed per day (categorical data)	r_s_ = -0.115	0.005*
Instant coffee	Number of times items consumed per day (categorical data)	r_s_ = -0.168	< 0.001*
Mate	Number of times items consumed per day (categorical data)	r_s_ = 0.019	0.64
Energy drinks	Number of times items consumed per week (categorical data)	r_s_ = -0.019	0.64
Natural fruit juice	Number of times items consumed per week (categorical data)	r_s_ = -0.014	0.74
Fruit	Number of times items consumed per week (categorical data)	r_s_ = -0.026	0.52
Vitamin C	Number of times items consumed per week (categorical data)	r_s_ = -0.007	0.87
Alcohol	Number of times items consumed per week (categorical data)	r_s_ = 0.077	0.06
Breakfast	Number of times items consumed per week (categorical data)	r_s_ = 0.033	0.41
Fast food	Number of times items consumed per week (categorical data)	r_s_ = -0.117	0.004*
Snacks	Number of times items consumed per week (categorical data)	r_s_ = -0.031	0.45
Scheduled eating (breakfast, lunch, dinner)	Categorical data (Yes; No)	t = -0.356	0.72
Cigarettes	Number of times items consumed per day (categorical data)	r_s_ = -0.026	0.53
Water pipe	Number of times items consumed per week (categorical data)	r_s_ = -0.067	0.10

In the multiple linear regression analysis, most variables studied in the initial Spearman's rank correlation analysis exhibited consistent patterns. For instance, daily consumption of tea (B = -0.334, 95% CI -0.618 – -0.049, *p* = 0.022) and instant coffee (B = -0.682, 95% CI -1.099 – -0.265, *p* = 0.001), along with the weekly consumption of fast food (B = -0.583, 95% CI -1.132 – -0.034, *p* = 0.038), were all negatively associated with students’ grades. Additionally, age was also found to be negatively associated with grades (B = -0.759, 95% CI -1.049 – -0.468, *p* < 0.001).

However, some differences became evident. Water pipe consumption exhibited a negative association with academic performance in the regression analysis (B = -0.700, 95% CI -1.396 – -0.005, *p* = 0.049), while traditional coffee consumption showed no significant association with grades (B = -0.033, 95% CI -0.346 – 0.280, *p* = 0.836). Details for the multiple linear regression analysis for students’ exam scores are provided in Table 4.

**Table 4:** Multiple linear regression analysis for students’ exam scores (*n* = 608, R^2^ = 12.8%, Max. VIF = 4.898)

**Variable (reference group in brackets)**	**Unstandardized Coefficients**				**95% CI for Beta**	
	**Beta**	**SE**	**β**	***p***	***LL***	***UL***
**Covariates**						
Residency type (Dormitory)						
Rented house	-0.782	0.839	-0.056	0.352	-2.429	0.866
Owned house	0.114	0.852	0.010	0.893	-1.558	1.787
Residency partners (Alone)						
Friends/Roommates	0.380	1.133	0.025	0.738	-1.846	2.606
Relatives	1.122	1.764	0.029	0.525	-2.343	4.587
Brothers/Sisters	1.385	1.318	0.060	0.294	-1.203	3.974
Family	1.426	1.034	0.118	0.168	-0.605	3.456
Sex (Female)	0.646	0.462	0.059	0.162	-0.261	1.553
Age	-0.759	0.148	-0.203	< 0.001*	-1.049	-0.468
**Variables of interest**						
Cigarettes	0.168	0.241	0.031	0.486	-0.305	0.641
Water pipe	-0.700	0.354	-0.080	0.049*	-1.396	-0.005
Traditional coffee	-0.033	0.159	-0.009	0.836	-0.346	0.280
Tea	-0.334	0.145	-0.095	0.022*	-0.618	-0.049
Instant coffee	-0.682	0.212	-0.142	0.001*	-1.099	-0.265
Mate	0.109	0.140	0.033	0.437	-0.166	0.384
Energy drink	-0.283	0.965	-0.012	0.770	-2.178	1.613
Natural fruit juice	-0.407	0.371	-0.045	0.273	-1.137	0.322
Fruit	0.122	0.232	0.022	0.600	-0.334	0.577
Vitamin C	0.033	0.326	0.004	0.920	-0.608	0.674
Alcohol	0.966	0.493	0.079	0.051	-0.004	1.935
Scheduled eating (No)	-0.613	0.469	-0.055	0.192	-1.534	0.309
Breakfast	0.074	0.101	0.032	0.465	-0.124	0.271
Fast food	-0.583	0.280	-0.092	0.038*	-1.132	-0.034
Snacks	0.027	0.208	0.005	0.897	-0.382	0.436

*CI* Confidence interval, *SE* Standard Error, *LL* Lower limit, *UL* Upper limit

^a^Dependent variable: Average exam scores from the last two semesters

## Discussion

The findings of this study provide valuable insights into the relationship between students’ eating, drinking, and smoking habits and their academic performance measured as average grades from the last two semesters. The study sheds light on healthy habits among medical students at Damascus University and may encourage them to adopt healthier lifestyles that can improve their academic performance.

The medical education program in Syria consists of a 6-year educational program that includes a pre-medical preparatory year, which is considered an alternative for the first year in the faculties of Medicine, Dentistry, and Pharmacy, followed by five years of study in basic and clinical sciences. The first 3 years are dedicated to basic sciences, and the 4th and 5th years focus on clinical content, while the 6th year is devoted to clinical training [15]. Each study year consists of two semesters, and at the end of every semester (except the sixth year), there is an exam period of approximately 1–2 months during which theoretical exams are conducted. These exams make up about 70–80% of the total grades that students receive every semester.

Regarding gender, our study found no differences in academic performance between males and females, which is consistent with previous studies [6, 16]. However, one study found that females had a higher GPA than males [17]. The absence of gender differences in academic performance may indicate a relatively equitable educational environment that doesn't favour one gender over the other. This suggests similar challenges and opportunities for both male and female students in this context.

Breakfast consumption has been shown to have a good impact on academic achievement among college students [5, 7, 8, 11, 16]. However, in our study, the consumption of breakfast during the exam period did not demonstrate a significant association with academic performance. These findings are consistent with a previous study, which similarly found no significant relationship between skipping breakfast and the cumulative average grades of medical students [18]. Considering regular meal intake, a previous systematic review observed a positive association between academic achievement and regular meal consumption [11]. However, that was not the case in our current study, as there was no statistical significance observed. This may be attributed to the limited scope of our study, as we only measured breakfast and regular meal consumption during the exam period without taking into account other factors such as the meal components and timing. Further research that includes these factors may provide more comprehensive insights.

Previous studies showed that alcohol consumption had a negative association with academic performance [7, 8, 17, 19, 20]. Whereas one study found no significant association with academic achievement [21], our study also yielded similar results, showing no significant relationship. The absence of a connection could be linked to the minimal alcohol consumption in our sample, potentially influenced by the prevalence of Muslim individuals. In nations with predominantly Muslim populations, religious and sociocultural factors often strongly discourage alcohol use [22, 23].

The prevalence of smoking cigarettes and water pipe in our study is nearly similar to a previous study conducted at the same faculty in 2008 [24]. In a previous study with Syrian adults, 15.9% of the sample had used a waterpipe before [25]. A waterpipe consists of a bowl with holes at the bottom, connected to a tube leading to a water container. Tobacco is heated in the bowl with hot charcoal, producing smoke that's inhaled through a hose [26]. Regarding the effect of smoking on academic achievement, some studies showed that lower academic achievement was associated with smoking [7, 10, 20, 27–29]. Our regression results revealed a statistically significant negative effect of waterpipe smoking, but no such effect was observed for cigarette smoking., The negative association could be due to various reasons, including the impact of smoking on cognitive function, concentration, or probably the time spent on smoking that could have otherwise been dedicated to studying. It's worth mentioning that we didn't include E-cigarettes in our study because accurately reporting the amount of E-cigarette consumption posed challenges.

While collecting the data, we asked about two forms of coffee-containing beverages: traditional coffee and instant coffee. Instant coffee is often preferred for its affordability and convenience when compared to traditional coffee. It typically contains lower levels of caffeine than traditional coffee [30]. However, it's worth noting that instant coffee has been found to contain approximately twice the amount of acrylamide as traditional coffee [31]. Excessive acrylamide intake is associated with potential toxic effects on the nervous system [32]. Additionally, consuming more than six cups of instant coffee per day has been linked to a higher risk of dementia [33]. Similarly, high daily tea consumption has also been associated with an increased risk of Alzheimer's disease [34].

After controlling for confounders, we found that lower students’ grades were associated with higher amounts of tea and instant coffee. In a previous systematic review, increased attention and alertness were noticed with lower doses of caffeine (< 4 g), whereas undesired side effects, such as anxiety, were noticed with higher amounts of caffeine [35]. However, previous studies observed that coffee consumption had no significant impact on academic achievement [20, 36]. Finally, it is important to note that our findings could be influenced by other factors such as ADHD, where individuals may consume larger quantities of caffeine. Therefore, the actual effect on academic performance could be attributed to these correlated factors.

In Syria, yerba mate is typically served in small 100 ml glasses, filled halfway with around 10 g of dry yerba mate leaves. A straw is used for drinking. Initially, cold water is used to soak the leaves, then it's replaced with hot water for steeping. This process is repeated for about an hour or more until the leaves are fully rinsed [37]. Yerba mate has shown no significant effects on cognitive performance [38]. Our study found no significant association between mate consumption during the exam period and academic achievement.

Energy drinks consumption has been negatively associated with academic achievement [8, 39–41], but in our study, no association was found. It is important to note that energy drinks consumption is not popular among the students in our sample; only 4.1% of the participants consumed energy drinks during the exam period.

Lower academic performance has been associated with not meeting fruit consumption recommendations [7, 11, 42], but other studies found no relation between the amount of fruit consumption and academic performance [5, 8], which matches our results.

Our findings regarding fruit juice consumption amount were consistent with previous studies [5, 8], as we also found no significant association with academic achievement.

In a prior study, a correlation was found between vitamin C plasma concentration and various aspects of cognitive performance. However, it is important to highlight that both vitamin C supplementation and sufficient vitamin C intake from dietary sources did not exhibit differential effects on cognitive performance [43]. In our study, we did not observe a significant association between the consumption amount of vitamin C tablet supplementation and students' grades. It is important to note that vitamin C can be obtained from various sources, and that could have interfered with our result, future research is needed to adjust for these confounding factors.

Finally, our findings are consistent with previous research indicating a negative association between fast food consumption and academic achievement [5, 7, 8, 44]. This may be attributed to the documented positive impact of various dietary components on cognitive function [4]. Conversely, the consumption of fast food, which is known to lack essential micronutrients, may potentially contribute to lower academic performance due to insufficient intake of these crucial nutrients [11]. This is further exacerbated by the additional effects of fast food consumption, such as overeating behaviour and feelings of fatigue [45].

Regarding snacks, a previous study found no significant association between snacks and academic achievement [46]. Similarly, we found no association between snack intake amount in the exam period and academic achievement. However, this result may be limited by the lack of detail in the survey question about what types of food are considered as snacks.

### Limitations

Our study had some limitations. First, it was conducted in a single university and may not be generalizable to other universities or populations. Second, the study was cross-sectional, which limits our ability to infer causality. In this context, it’s important to note that the observed correlations may not solely be due to the impact of these habits during the exam period, as these habits could extend beyond this timeframe. Third, our study relied on self-reported data, which may be subject to recall bias and social desirability bias. For future research, longitudinal studies should be conducted to establish a causal relationship between dietary habits and academic performance and compare the effects of these habits both inside and outside of the exam period. Additionally, multicenter studies could be conducted to increase the generalizability of the findings beyond a single university.

## Conclusions

Our results indicated a significant negative correlation between medical students’ grades and their consumption amount of tea, instant coffee, and fast food. However, it's important to note that the correlation was weak. These findings emphasize the need for further awareness campaigns and health education programs to promote healthy habits among medical students during exams. Future studies could further explore the impact of other lifestyle factors on academic performance among medical students, as they may have a bigger impact.

### Supplementary Information

**Additional file 1.**

## Data Availability

The datasets used and/or analysed during the current study are available from the corresponding author [AB] on reasonable request.
