# Supplementary material for: Dietary and smoking habits during the exam period and their effect on the academic achievement among Syrian medical students
Source: BMC Med Educ. 2024 Jan 12;24:60. doi: 10.1186/s12909-023-04950-6 (PMC10785519; doi:10.1186/s12909-023-04950-6)
Supplement: Supplementary file 1 — Additional file 1. [file 12909_2023_4950_MOESM1_ESM.docx]

**Eating, Drinking, and Smoking Habits during the exam period, and its relation to students marks among medical students at Damascus University**

In this survey, information about dietary habits and students’ marks will be collected:  agree  disagree

- **Gender:**  Male  Female
- **Age** _ _ _ _
- **Year of study:**  Second  Third  Fourth  Fifth  Sixth
- **Residency type:**  Dormitory  Rented House  Owned House
- **I live:**  With family  With brothers/sisters  With relatives  With roommates/friends  Alone

NOTE: For second-year students, please provide your average score in the preparatory year (without adding your score in the 12^th^ grade)

- **What was your average score in the last two semesters?** _ _ _ _
- **Are you a smoker?**  Water pipe + Cigarettes  Cigarettes only  Water pipe only  I used to smoke before  I never smoked
- **How many Cigarettes do you smoke on an average day?** 0 1-5 6-10 11-20 21-30  More than 31
- **How many Cigarettes do you smoke per day during the exam period?** 0  1-5 6-10 11-20 21-30  More than 31
- **How often do you smoke Water pipe typically?** Never Once a week Two or more times a week Once daily More than once daily
- **How often do you smoke Water pipe during the exam period?** Never Once a week Two or more times a week Once daily More than once daily
- **During the exam period,** how many cups of traditional coffee do you typically drink **per day?** 0 (I don’t drink) 1 2 3 4 5 or more
- **During the exam period,** how many cups of tea do you typically drink **per** **day?** 0 (I don’t drink) 1 2 3 4 5 or more
- **During the exam period,** how many cups of Instant coffee (e.g. Nescafe/Cappuccino) do you typically drink **per day?** 0 (I don’t drink) 1 2 3 4 5 or more
- **During the exam period,** how many times do you typically drink Mate **per** **day?** (Refill the cup)0 (I don’t drink) 1 2 3 4 5 or more
- **During one week of the exam period,** how many beverages (250ml) of energy drinks do you drink typically? (I don’t drink) 1-3 4-6 7 or more
- **During one week of the exam period,** how many natural fruit juice cups do you typically drink? 0 (I don’t drink) 1-3 4-6 7 or more
- **During one week of the exam period,** how often do you eat fruit (excluding fruit juice)? Rarely 1-2 3-4 Daily
- **During one week of the exam period,** how often do you consume vitamin C tablets? 0(Never) 1-3 4-6 7-10 11 or more
- **During one week of the exam period,** how many alcoholic beverages do you consume? 0(I don’t drink)  1 2-3 4-6 7-9 10 or more
- **During one week of the exam period,** do you follow a scheduled eating pattern (breakfast- lunch- dinner)? Yes No
- **During one week of the exam period,** how many days a week do you have breakfast? 0(Never) 1 23 4 56 7(every day)
- **During one week of the exam period,** how many times do you consume fast food meals? Rarely 1-2 3-4 Every day
- **During one week of the exam period,** how many times do you consume snack meals? Rarely 1-2 3-4 Every day
